# Formulation of a Stable Oil-in-Water Microemulsion of *Torreya grandis* cv. Merrillii Aril Essential Oil and Its Application in Loquat Fruit Preservation

**DOI:** 10.3390/foods12214005

**Published:** 2023-11-01

**Authors:** He Wang, Yue Zheng, Xinyue Tang, Ting Zhang

**Affiliations:** 1 School of Grain Science and Technology, Jiangsu University of Science and Technology, Zhenjiang 212100, China; 18796012161@163.com; 2 Jiyang College, Zhejiang Agriculture and Forestry University, Shaoxing 311800, China; zhangting9177@163.com; 3 Faculty of Food Science and Engineering, Kunming University of Science and Technology, Kunming 650500, China; zheng_yue21@163.com

**Keywords:** microemulsion, *Torreya grandis* cv. Merrillii aril, essential oil, loquat, fruit quality

## Abstract

Loquat is a nutrient-rich fruit with juicy and sweet pulp, but it is vulnerable to rot and deterioration without proper postharvest preservation measures. This study aimed to improve the postharvest quality of loquat by developing a microemulsion system based on an essential oil extracted from the *Torreya grandis* cv. Merrillii aril (TaEO), which has antimicrobial and antioxidant properties. An optimal TaEO microemulsion (TaEO-ME) was formulated, using a mixture of Tween-40 and Tween-80 as the surfactant, 1-butanol as the co-surfactant, and TaEO as the oil phase, with mass ratios of 9:1, 3:1, and 6:1, respectively. Two TaEO-ME formulations with 60% and 70% water contents were stable for 180 days at room temperature, with a mean droplet size below 12 nm and polydispersity index less than 0.24. They also exhibited higher stability and enhanced biological activities compared to free TaEO. Loquat fruit treated with TaEO-ME displayed a reduced decay index and lower membrane lipid peroxidation after 15 days of storage at 15 °C, as indicated by the lower malondialdehyde content and higher peroxidase activity. Moreover, the TaEO-ME treatment preserved the nutrient quality by maintaining the total phenolic compounds and ascorbic acid content. Our findings suggested that TaEO-ME can be used as a substitute for chemical preservatives to keep fruits fresh.

## 1. Introduction

Loquat (*Eriobotrya japonica* Lindl.) is a fruit crop native to China that has high nutritional and enjoyable sensory qualities. However, its postharvest life is limited by its susceptibility to mechanical injury, physiological disorders, and microbial infection, which are attributed to its thin peel, tender flesh, and fragile stalk. To prolong the shelf life of fruits, synthetic preservatives have been widely used to inhibit the growth of various fungal pathogens [1]. Nevertheless, the excessive use of these chemicals poses serious threats to the environment and human health, raising concerns globally [2]. Therefore, there is an urgent need to develop effective natural preservatives. Essential oils, which are natural plant extracts with health benefits, may alleviate concerns about food safety and quality by serving as potential alternatives to synthetic preservatives.

*Torreya grandis* cv. Merrillii (Taxaceae) is an economically valuable tree that produces dried nuts with high nutritional and medicinal value in China [3]. The industrial processing of the nuts generates a large amount of aril, which is an underutilized by-product that causes environmental problems and is a bioresource waste. This by-product contains various bioactive components such as volatile oils, flavonoids, and taxols [4]. Recently, the essential oil extracted from the aril of *Torreya grandis* cv. Merrillii (TaEO) was shown to have good antioxidant, antibacterial, and tyrosinase-inhibiting activities, which indicate its potential for application in food and skincare products [5]. However, the incorporation of essential oils like TaEO into real food systems is challenging due to their poor water solubility, high volatility, and chemical instability against light, heat, and oxygen [6]. Therefore, it would be highly desirable to enhance the stability of the volatile components in essential oils while preserving the overall efficacy of essential oils. One of the promising technologies in this area is microemulsion, which is a transparent, isotropic, and thermodynamically stable colloidal dispersion of oil droplets in water, stabilized by a surfactant and co-surfactant [7]. It has been reported that essential oils in a microemulsion form exhibit improved antimicrobial and antioxidant properties, as well as enhanced water solubility, compared to their non-emulsified counterparts. Furthermore, microemulsions of essential oils have shown a potential to preserve the quality and extend the storage life of various fruits and vegetables, such as leach fruit and iceberg lettuce [8,9]. Hence, the microemulsions of essential oils have promising potential to be applied as natural preservatives for fresh produce.

To the best of our knowledge, the literature on the use of micro-emulsified TaEO for food preservation is limited. The aim of this study was therefore to fabricate oil-in-water (O/W) microemulsions of TaEO (TaEO-ME) using the pseudo-ternary phase diagram method and to characterize their physicochemical properties, such as electrical conductivity, viscosity, droplet size, ζ-potential, and storage stability. The in vitro antioxidant, antityrosinase, and antibacterial activities of TaEO and TaEO-ME were also evaluated. Furthermore, the potential use of TaEO-ME as a natural preservative for loquat fruit was investigated by evaluating its effects on the qualities of the fruit during storage.

## 2. Materials and Methods

### 2.1. Materials and Chemicals

Tween-20, Tween-40, Tween-80, Span-80, anhydrous ethanol, and glycerol were of analytical grade and purchased from Sinopharm Chemical Reagent Co., Ltd. (Shanghai, China). 1-Butanol was supplied by Tianjin Zhiyuan Chemicals Co., Ltd. (Tianjin, China). 2,2-Diphenyl-1-picrylhydrazyl radical (DPPH) and 2,2’-azo-bis (3-ethylbenzothiazole-6-sulfonic acid) (ABTS) were purchased from Sigma-Aldrich (Shanghai) Trading Co., Ltd. (Shanghai, China). Tyrosinase (derived from mushrooms, 500 U/mg) was purchased from Shanghai Ruiyong Biotechnology Co., Ltd. (Shanghai, China). Levodopa (L-DOPA, purity ≥ 99.0%) was bought from Braunway Technology Co., Ltd. (Beijing, China). 1-(4-(phenylzao)phenyl)azo-2-naphthol (Sudan III) was purchased from Shanghai Aladdin Biochemical Technology Co., Ltd. (Shanghai, China). TaEO was prepared according to the published protocol in our previous report [10]. The bacterial strains used in this study were *Staphylococcus aureus* (ATCC29213), *Listeria monocytogenes* (ATCC19115), *Shigella castellani* (ATCC25931), *Salmonella typhimurium* (ATCC14028), and *Escherichia coli* (ATCC25922), which were acquired from Shanghai Luwei Technology Co., Ltd. (Shanghai, China). 

### 2.2. Preparation of TaEO-ME and Construction of the Pseudo-Ternary Phase Diagram

#### 2.2.1. Preparation of TaEO-ME

A pseudo-ternary phase diagram was constructed to determine the concentration range of each component. The water titration method was employed to prepare TaEO-ME by diluting the surfactant/TaEO mixture with water. Based on the preliminary screening experiments of the surfactant and co-surfactant, a combination of Tween 40 and Tween 80 at a weight ratio of 9:1 was selected as the surfactant and 1-butanol was used as the co-surfactant. The surfactant mixture was obtained by mixing the surfactant and 1-butanol at different mass ratios (*K_m_*, surfactant to co-surfactant) of 1:1, 2:1, 3:1, and 4:1. Then, TaEO was added to the surfactant mixture at a predetermined mass ratio (*S_m_*, surfactant mixture to oil) of 4:1, 5:1, 6:1, 7:1, 8:1, and 9:1. Next, water was added dropwise into the prepared self-emulsifying system under moderate agitation until the system transitioned from turbid to clear. Meanwhile, the amount of distilled water added was recorded and the percentage of each component in the phase diagram was calculated. The TaEO-ME region was identified and marked in the phase diagrams using Origin software version 8.5 (Origin Lab Corporation, Northampton, MA, USA).

#### 2.2.2. Effects of Ionic Strength and pH on the Formation of TaEO-ME

The influence of ionic strength on the microemulsion zone was evaluated by using NaCl solutions of 1, 2, and 3 mol/L as the aqueous phase instead of deionized water. Likewise, the effect of pH (5, 7, and 9) on the microemulsion zone was examined by using disodium hydrogen phosphate-potassium hydrogen phosphate-buffered solution as the aqueous phase for the microemulsion preparation. 

### 2.3. Type Identification of TaEO-ME via Electrical Conductivity

The electrical conductivity method was applied to identify the type of microemulsion achieved with various water contents. A DDS-307A conductivity meter (Rex Instruments Factory, Shanghai, China) was employed to perform the electrical conductivity measurements. The electrode was immersed in the microemulsion sample until equilibrium was reached. The data were recorded when the reading became stable. The curve of the electrical conductivity as a function of water content was obtained using Origin 8.5 software.

### 2.4. Characterization of TaEO-ME with 60% and 70% Water Content

#### 2.4.1. Differential Scanning Calorimetry (DSC) Measurement

The thermal behavior of TaEO-ME was investigated via DSC analysis using a DSC 214 Polyma (NETZSCH, Selb, Germany). TaEO-ME (10 mg) was accurately weighed and sealed hermetically into a standard aluminum crucible immediately. The crucible was quickly cooled from ambient temperature to −40 °C. After 20 min of equilibration, the sample was heated from −40 °C to 40 °C at a rate of 5 °C/min. An empty crucible was treated as a reference.

#### 2.4.2. Storage Stability

The stability of the two microemulsions was evaluated after 0 days and 180 days of storage at room temperature. The polydispersity index (PDI), particle size, and ζ-potential of the microemulsion were determined using a Zetasizer Nano ZS90 (Malvern Instruments Ltd., Malvern, UK) after a ten-fold dilution with distilled water.

#### 2.4.3. Rheological Properties

The rheometer (MCR 102 Anton Paar, Anton Paar GmbH, Graz, Austria) was used to measure the viscosity (diameter 40 mm, gap 1 mm) of 60% and 70% TaEO-MEs at 20–45 °C. The fixed shear rate was 50 s^−1^, and the relationship between the apparent viscosity (mPa·s) and temperature (°C) of the microemulsion was determined at a heating rate of 5 °C/min. The temperature scan range was 20 to 45 °C.

### 2.5. Antioxidant Activity of TaEO-ME with 60% and 70% Water Content

#### 2.5.1. ABTS Measurement

The scavenging ability of TaEO and TaEO-ME against ABTS•^+^ radical was evaluated via Sun et al.’s method [11] with slight modifications. Briefly, 7 mM of ABTS and 2.45 mM potassium persulfate were mixed in an equal volume ratio. After being kept in the dark for 12 h, the mixture was diluted with methanol to obtain the ABTS•^+^ solution with an absorbance of approximately 0.7 at 745 nm. Then, 0.3 mL of TaEO or TaEO-ME dissolved in methanol was added into 2.7 mL of the prepared ABTS•+ solution and incubated at room temperature in the dark for 6 min. The absorbance of the reaction mixture at 745 nm was immediately measured using a SpectraMax M5 microplate reader (Molecular Device, Sunnyvale, CA, USA). The free radical scavenging activity of ABTS can be calculated using the following formula:

(1) $$\mathrm{ABTS}\;\mathrm{radical}\;\mathrm{scavenging}\;\mathrm{activity}\;(\%)=\frac{A_{c}\;-\;A_{s}}{A_{c}}\;\times \;100$$

where *A_c_* is the absorbance of methanol mixed with ABTS•^+^; *A_s_* is the absorbance of the sample mixed with ABTS•^+^.

#### 2.5.2. DPPH Measurement

The DPPH free radical scavenging ability of TaEO and TaEO-ME was assessed following Sun et al.’s method [11] with minor modification. In brief, 0.5 mL of TaEO or TaEO-ME prepared in methanol was added to 2.0 mL of 0.1 mM DPPH solution. The resulting mixture was then allowed to stand for 30 min in dark and its absorbance was recorded at 517 nm with a SpectraMax M5 microplate reader (Molecular Device, Sunnyvale, CA, USA). The scavenging activity of DPPH free radical can be computed using the following equation:

(2) $$\mathrm{DPPH}\;\mathrm{radical}\;\mathrm{scavenging}\;\mathrm{activity}\;(\%)=\frac{A_{1}\;-\;A_{2}}{A_{1}}\;\times \;100$$

where *A*_1_ is the absorbance of a mixture of methanol and DPPH; *A*_2_ is the absorbance of the sample mixed with DPPH.

### 2.6. Tyrosinase Inhibitory Effect of TaEO-ME with 60% and 70% Water Content

The inhibition of tyrosinase by TaEO and TaEO-ME was measured using a modified version of a previously reported method [12]. In brief, 80 μL of PBS (0.1 M, pH 6.8) was dispensed into each well of a 96-well plate, followed by the addition of 40 μL of the sample solution and 40 μL of 3 mM levodopa (L-DOPA). The plate was incubated at 37 °C for 10 min, and then 40 μL of tyrosinase (100 U/mL) was added to each well to initiate the reaction. After another incubation at 37 °C for 30 min, the absorbance of the reaction mixture was measured at 475 nm using a SpectraMax M5 (Molecular Device, Sunnyvale, CA, USA) microplate reader. The percentage of tyrosinase inhibition was calculated as follows:

(3) $$\mathrm{Tyrosinase}\;\mathrm{inhibition}\;(\%)=\frac{A_{3}\;-\;A_{4}}{A_{3}}\;\times \;100$$

where *A*_3_ is the absorbance of the control that contained all the components without L-DOPA and *A*_4_ is the absorbance of the sample.

### 2.7. Antibacterial Activity of TaEO-ME with 60% and 70% Water Content

The antibacterial activity of TaEO and TaEO-ME was evaluated using the Oxford cup method described by Diao et al. [13] with a slight modification. Briefly, the essential oil was sterilized via filtration through a 0.22 µm microporous filter. *S. aureus* (ATCC29213), *L. monocytogenes* (ATCC19115), *S. castellani* (ATCC25931), *S. typhimurium* (ATCC14028), and *E. coli* (ATCC25922) were cultured on a nutrient broth medium at 37 °C for 12 h (the same conditions were used for the subsequent tests). Then, 200 µL of each bacterial suspension at a concentration of 1 × 10^6^ CFU/mL was spread on the nutrient agar medium. A sterile Oxford cup (8 mm in diameter) was placed on the surface of inoculated agar medium, and a volume of 200 µL of TaEO or TaEO-ME was added immediately into the Oxford cup. A control sample containing the mixed surfactants was also tested. Finally, the plates with the Oxford cup were incubated at 37 °C for 24 h, and the diameter of the inhibition zone was measured.

### 2.8. The Fresh-Keeping Ability of TaEO-ME on Loquat Fruit

#### 2.8.1. Loquat Material and Treatment

The loquat fruits (cv. “Baiyu”) used in this study were hand-harvested from a commercial orchard in Zhenjiang, Jiangsu province, China, with approximately 1 cm of the stems attached. The fruits were graded for size, color, and maturity, and discarded if they had any decay, insect damage, or mechanical injury. The selected fruits were randomly divided into four groups and immersed for 2 min in different solutions: (1) sterile water (designated as Control-Water), (2) a mixed solvent containing all the components of 0.1 mg/mL TaEO-ME except for the essential oil (Control-ME), (3) 0.1 mg/mL TaEO solution (TaEO), and (4) 0.1 mg/mL TaEO-ME solution (TaEO-ME). The optimal concentration of TaEO (0.1 mg/mL) for loquat fruit preservation was determined via a preliminary screening experiment. After immersion, the fruits were drained and air-dried on a sterile bench. Twenty fruits per group were packed in round plastic boxes (15 cm × 15 cm) covered with perforated cling film and stored at 15 ± 1 °C and 80% RH for 15 days. The quality parameters and enzyme activities of the fruits were measured at 0, 3, 6, 9, 12, and 15 d of storage.

#### 2.8.2. Determination of Quality Indicators

The decay index and ascorbic acid content of the loquat fruits were measured according to the method described by Wang et al. [10].

The total phenolic content of loquat fruit was determined using the Flion-Ciocalteu reagent with some modifications, as reported by Wang et al. [14]. Four grams of peeled loquat pulp was mixed with 10 mL of 80% (*v*/*v*) methanol solution containing 0.2% formic acid (*v*/*v*) and refrigerated for at least 30 min. The mixture was then ground and homogenized in an ice bath to extract the phenolic compounds from the pulp. The homogenate was centrifuged at 4 °C, 12,000 rpm for 20 min and the supernatant was collected. A mixture of 50 µL of supernatant, 150 µL of distilled water, 1 mL of Flion-Ciocalteu reagent, and 0.8 mL of 75 g/L sodium carbonate solution was prepared and incubated in a water bath at 30 °C for 1 h. The absorbance value was measured at 765 nm. The total phenolic content was expressed as gallic acid equivalents (GAE) in mg per 100 g of fresh weight.

The malondialdehyde (MDA) content, an indicator of lipid peroxidation, was measured using a MDA Assay Kit (Nanjing Jiancheng Bioengineering Institute, Nanjing, Jiangsu, China). Loquat fruit tissue was homogenized in cold saline (0.86%, 1:2 g/mL) and centrifuged to obtain the supernatant. A volume of 0.2 mL of the supernatant was reacted with thiobarbituric acid and the absorbance was read at 532 nm. The MDA content was calculated as nmol/mg protein.

#### 2.8.3. Determination of the Activities of Defense-Related Enzymes

The activities of catalase (CAT), peroxidase (POD), and polyphenol oxidase (PPO) in the loquat fruits were detected using the method described by Wang et al. [10]. The enzyme extracts were prepared from fresh loquat pulp and analyzed using specific commercial kits provided by Nanjing Jiancheng Bioengineering Institute (Jiangsu, China), in accordance with the manufacturer’s guidelines.

### 2.9. Statistical Analysis

Each experiment was replicated three times, and the results were reported as mean (*n* = 3) ± standard deviation (SD). The data were checked for normality (Shapiro–Wilk test) and homogeneity of variance (Levene’s test). The significance of the difference (*p* < 0.05) was assessed via one-way analysis of variance with Tukey’s post hoc test. All analyses were performed using Origin software 8.5.

## 3. Results and Discussion

### 3.1. Optimization of the TaEO-ME Formulation

#### 3.1.1. Screening of Surfactants and Co-Surfactants

The hydrophilic–lipophilic balance (HLB) of the surfactant’s and the co-surfactant’s type is a key factor that affects the stability of emulsions [15]. Generally, a HLB value of 8–18 is favorable for the formation of an oil-in-water microemulsion. In this study, a mixture of Tween-40 + Tween-80 at a mass ratio of 9:1 and 1-butanol were employed as the surfactant and co-surfactant, respectively, for microemulsion preparation. Tween (polysorbate) is a non-ionic surfactant that has a wide range of applications as an emulsifier in food, cosmetic, and pharmaceutical products due to its relatively high safety profile. 1-butanol is a valuable platform chemical that can be produced by the anaerobic continuous acetone-butanol-ethanol fermentation of glucose derived from pretreated biomass. It is widely used in the chemical, pharmaceutical, and food industries as a green solvent [16]. The combination of surfactants was found to enhance the emulsification of essential oils, which may be attributed to the increased surfactant partitioning at the oil–water interface [17]. The role of the co-surfactant is to lower interfacial tension and adjust the HLB of the surfactant. 1-butanol possesses both hydrophilic and lipophilic properties, which can modify the interfacial spontaneous curvature and the flexibility of the interface layer, and regulate the HLB value to improve the oil solubilization capacity of the microemulsion.

#### 3.1.2. Effect of the Surfactant/Co-Surfactant Mass Ratio (K_m_) on TaEO-ME Formation

**Figure 1:**
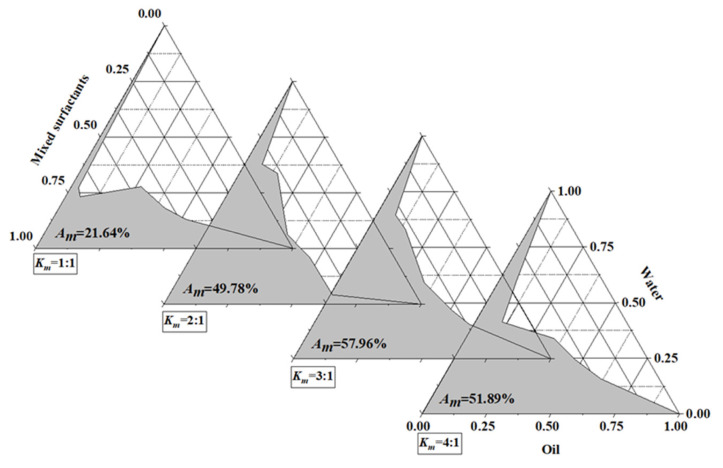
Pseudo-ternary phase diagram of *Torreya grandis* cv. aril essential oil microemulsion with different *K_m_*s. The shaded areas represent the transparent monophasic microemulsion regions and are denoted as *A_m_*.

The optimal combination of surfactant and co-surfactant that yielded the largest microemulsion area was identified by constructing pseudo-ternary phase diagrams. Then, the influence of *K_m_* on the formation of TaEO-ME was investigated. The TaEO-ME was formulated by blending Tween-40 + Tween-80 (9:1) with 1-butanol at different *K_m_* values of 1:1, 2:1, 3:1, and 4:1, respectively, and the corresponding pseudo-ternary phase diagrams were plotted. As shown in Figure 1, the area of the microemulsion zone varied with *K_m_* in the sequence of 3:1 > 4:1 > 2:1 > 1:1. The results showed an increase in the TaEO-ME region area from 21.64% to 57.96% as *K_m_* values increased from 1:1 to 3:1, but a further increment of surfactant concentration led to a decrease in the TaEO-ME area (51.89%). This was probably due to the inadequate concentration of co-surfactant, which makes it impossible to enhance the stability of TaEO-ME by lowering the interfacial tension and the rigidity of the interface [18]. Therefore, the optimal *K_m_* value was 3 for TaEO-ME formation.

#### 3.1.3. Effect of the Surfactant/TaEO Mass Ratio (S_m_) on TaEO-ME Formation

The mass ratio of mixed surfactant to TaEO (*S_m_* = 4:1, 5:1, 6:1, 7:1, 8:1, and 9:1) was varied to investigate its effect on the formation of TaEO-ME. The content of the aqueous phase in the microemulsion was fixed at 90% to examine the physical characteristics of TaEO-ME such as particle size, PDI value, and ζ-potential. As shown in Table 1, the average particle size decreased steadily with an increasing *S_m_* value, but the particle sizes at 4:1 and 5:1 were 143.90 nm and 109.83 nm, respectively, which were significantly bigger than that of a typical microemulsion (10–100 nm). The higher ratios (*S_m_* = 6:1, 7:1, 8:1, and 9:1) gave smaller particle sizes, which were 13.00, 12.38, 11.67, and 11.36 nm, respectively. Moreover, all the formulations except at 4:1 and 5:1 had a relatively low PDI value (0.19–0.26) and high absolute value of their ζ-potential, indicating a narrow droplet size distribution. These results demonstrated that the mixed surfactant/TaEO mass ratio of ≥6:1 was suitable for preparing stable microemulsions. Importantly, a high essential oil content in the microemulsion is preferred for practical use. As a result, the ideal *S_m_* value for microemulsion formulation was determined to be 6:1.

#### 3.1.4. Effects of Ionic Strength and pH on TaEO-ME Formation

**Figure 2:**
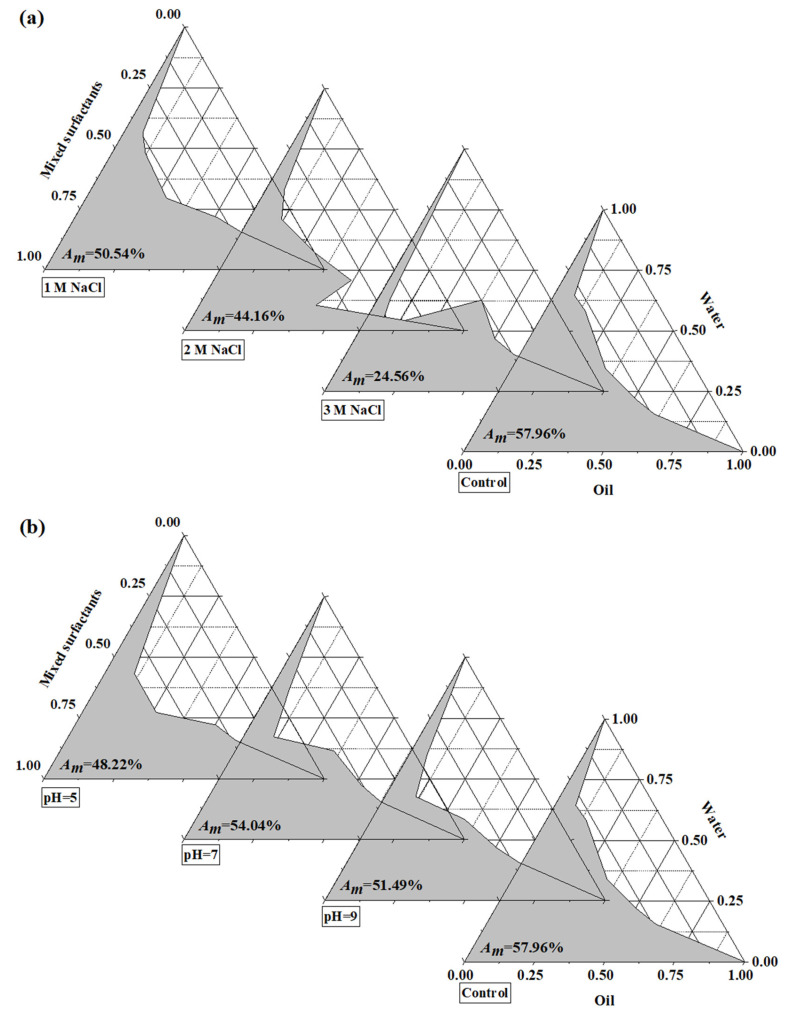
Effect of various concentrations of NaCl (**a**) and pH (**b**) on the one-phase areas of *Torreya grandis* cv. aril essential oil microemulsion. The regions where the microemulsion is transparent and consists of a single phase are shaded in the diagram and labeled as *A_m_*.

The presence of electrolyte was hypothesized to alter the microemulsion region in the phase diagram by influencing the droplet size of the dispersed phase [19]. Figure 2 shows that the area of the TaEO-ME region was affected by both NaCl and pH, with NaCl having a larger effect than pH. As the NaCl concentration increased, the microemulsion area decreased from 57.96% to 24.56%, suggesting that the surfactants were salted out of the microemulsion at a higher concentration of NaCl, and thereby reduced its hydrophilicity and emulsifying ability. The emulsification was also impaired at both low and high pH values, with acidic conditions being more detrimental than alkaline ones. Therefore, TaEO-ME formation and stability were favored in low-salt and neutral or slightly alkaline conditions.

### 3.2. Electrical Conductivity Analysis

**Figure 3:**
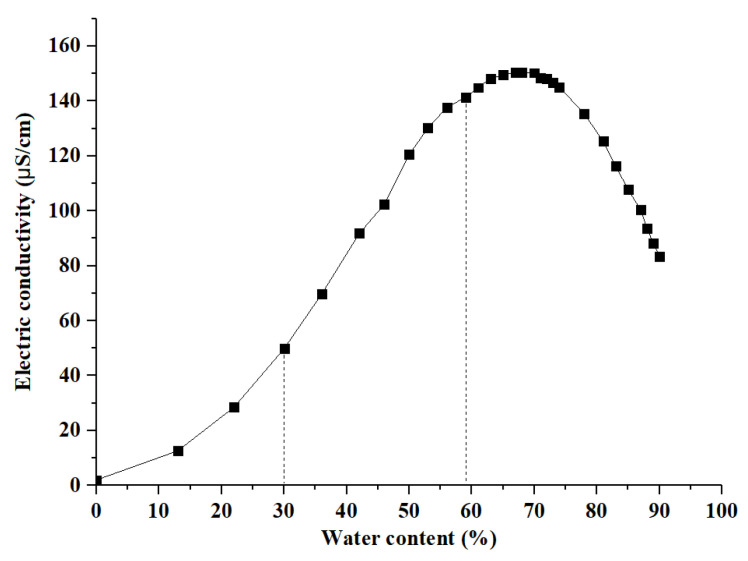
Electrical conductivity of *Torreya grandis* cv. aril essential oil microemulsion with the increase in water content.

The relationship between electrical conductivity and water volume fraction in the microemulsion can be used to analyze the structure type of the microemulsion. Figure 3 presents how the electrical conductivity of TaEO-ME varies with different water volume fractions. The curve exhibits a bell-shape profile, indicating a structural transition from W/O to O/W microemulsion. In the range of 0–30% water content, the electrical conductivity of microemulsions increases slowly with increasing water content. This is because the surfactant and oil phase had weak electrical conductivity. On the other hand, water molecules are encapsulated in reverse micelles and have limited contact with each other, which results in the low electrical conductivity of the mixture. The microemulsion structure in this range was W/O-type. In the range of 30–60% water content, the electrical conductivity of the microemulsion increases sharply with increasing water content, as water molecules are released from the reverse micelle and form a continuous conductive chain [20]. The microemulsion structure in this range was bi-continuous-type. As the water content increases from 60 to 90%, the electrical conductivity of the microemulsion exhibits a slow rise followed by a gradual decline, with a peak at 70% water content. This can be explained by the formation of a conductive chain, which reaches its optimal state at 70% water content. Beyond this threshold, the further increase in water content has a diluting effect that lowers the electrical conductivity of the microemulsion. The microemulsion structure in this range was of an O/W type. Similar microstructural transitions have also been observed in microemulsions with different oils such as sacha inchi oil [21] and tomato seed oil [22].

### 3.3. Characterization of TaEO-ME with 60% and 70% Water Content

O/W microemulsions are an efficient delivery carrier for incorporating lipophilic bioactive components such as essential oils into aqueous media and have been widely utilized in various fields, including food, pharmaceuticals, and cosmetics [23]. The encapsulation of lipophilic bioactive compounds into O/W microemulsions may enhance their aqueous compatibility, and retard their chemical degradation as well as increase their bioactivity [24]. Furthermore, the formulation of O/W microemulsions has to comply with the regulatory and economic requirements of the food industry. TaEO-MEs with a water content of 60% and 70% were chosen as the optimal microemulsion formulation for further investigation.

#### 3.3.1. DSC Analysis

**Figure 4:**
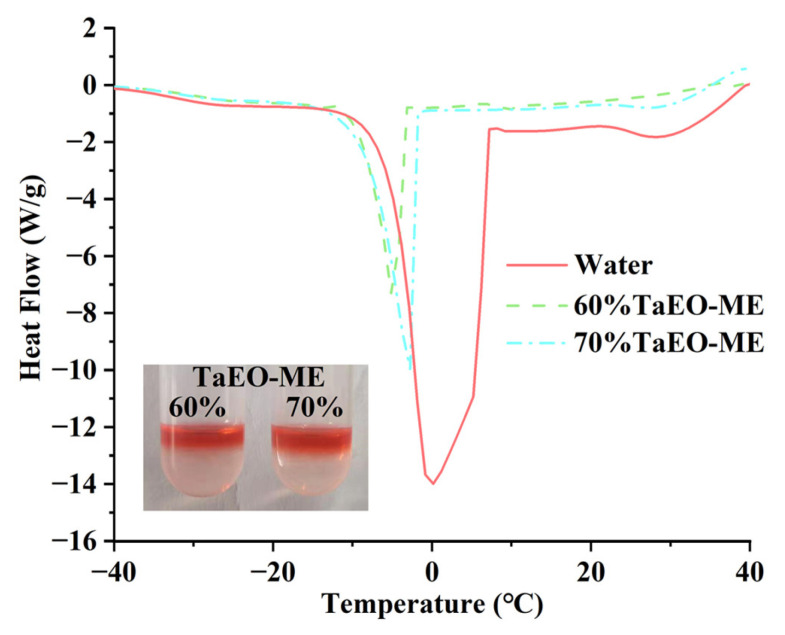
DSC heating curves of *Torreya grandis* cv. aril essential oil microemulsions.

DSC analysis has emerged as a powerful tool for exploring the microstructure and water behavior of microemulsions. Based on the study by Senatra et al. [25], we know that the water in the microemulsion system can exist in three distinct states: (i) free water, which melts at around 0 °C; (ii) interface water, which refers to the water at the interface of the dispersion system with a melting point of about −10 °C; and (iii) bound water, which is associated with the hydrophilic groups and usually melts below −10 °C. Figure 4 shows the DSC diagrams of 60% and 70% TaEO-MEs and ultrapure water. Both 60% and 70% TaEO-MEs exhibited a prominent endothermic peak near 0 °C. Moreover, the endothermic peak of TaEO-ME shifted to higher temperature as the water content increased from 60% to 70%. This shift might indicate that, when more water was added to the 60% TaEO-ME, free water became the predominant form of water. It was noteworthy that the peak temperature rose from −5.14 °C to −2.78 °C with the increasing water content, which approached the melting point of free water at 0 °C. The DSC results suggest that TaEO-MEs with 60% and 70% water content are O/W-type microemulsion systems.

At the same time, the dyeing diffusion experiment was performed on 60% and 70% TaEO-MEs (Figure 4). The type of microemulsion can be intuitively judged by observing the diffusion behavior of the oil-soluble dye Sudan red III in the microemulsion system. A uniform distribution of Sudan red III throughout the microemulsion indicates a W/O microemulsion; on the contrary, if Sudan red III floats on the surface of the microemulsion, it implies an O/W type; a state between the two suggests a bi-continuous microemulsion. In this study, Sudan red III was found to be completely insoluble and floated on the upper layer of the two formulations of TaEO-ME. The staining method further verified the oil-in-water nature of both 60% and 70% TaEO-MEs, which was consistent with the result of the DSC analysis.

#### 3.3.2. Storage Stability

**Figure 5:**
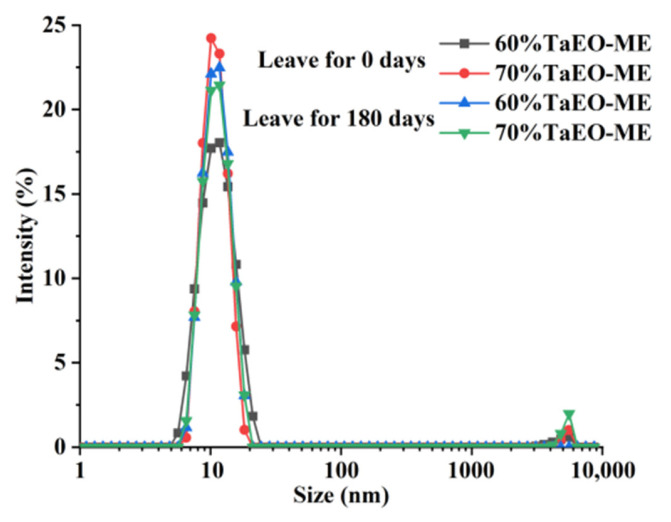
Particle size distribution of *Torreya grandis* cv. aril essential oil microemulsion.

The storage stability of microemulsion stored for 0 and 180 days was evaluated by measuring the droplet size, PDI, and ζ-potential using a particle size analyzer. The results are shown in Table 2 and Figure 5. The mean droplet sizes of TaEO-MEs with 60% and 70% water content after 0 and 180 days at room temperature were within the range of 10 to 12 nm, which is in agreement with the definition of microemulsions as thermodynamically stable and isotropic dispersions of water, oil, and surfactant, with droplet diameters of between 1 and 100 nm, and typically 10 to 50 nm [26]. Moreover, no significant difference in the droplet size distribution of the two microemulsions was observed after 180 days of storage compared to the initial measurement (*p* > 0.05), indicating that both microemulsions remained stable over time. The PDI values of 60% and 70% TaEO-ME were also below 0.3, which indicates a relatively narrow and uniform distribution of droplets in the aqueous phase [27]. There was no significant change in the PDI values between the initial and final measurements (*p* > 0.05). In addition, the ζ-potentials of the 60% and 70% TaEO-MEs decreased remarkably from −0.21 and −0.45 to −1.50 and −1.72 mV, respectively. Such a low ζ-potential can be ascribed to the non-ionic nature of the surfactant (Tween 40 and Tween 80) and co-surfactant (1-butanol) used in this study. Low ζ-potential can enhance the stability of a microemulsion by reducing electrostatic repulsion between droplets. Based on these results, 60% and 70% TaEO-MEs with a favorable particle size, PDI, and ζ-potential were obtained.

#### 3.3.3. Rheological Properties

**Figure 6:**
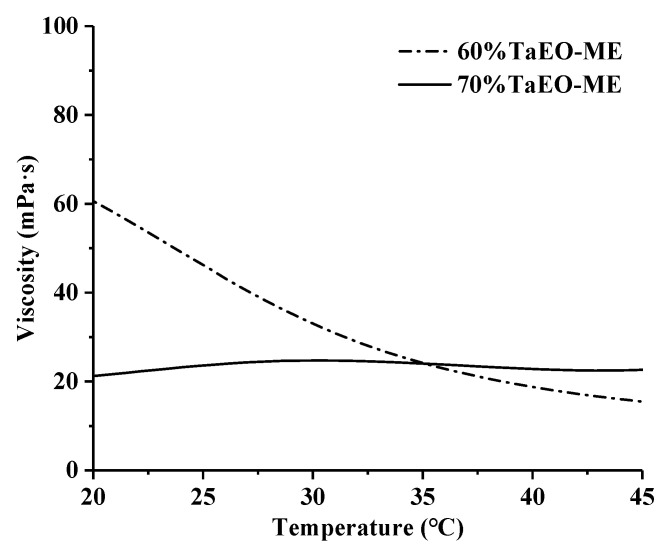
Viscosity changes of *Torreya grandis* cv. aril essential oil microemulsions at different temperatures. 60% TaEO-ME and 70% TaEO-ME represent microemulsions with water contents of 60% and 70%, respectively.

The change in viscosity of 60% and 70% TaEO-MEs with increasing temperature was measured using a rheometer, and the results are shown in Figure 6. The viscosity of 70% TaEO-ME was 21.39 mPa·s at 20 °C and 22.69 mPa·s at 45 °C, showing no significant change with increasing temperature. However, the viscosity of 60% TaEO-ME decreased markedly from 60.29 mPa·s to 15.44 mPa·s as the temperature increased from 20 to 45 °C, indicating that temperature had a pronounced influence on its rheological behavior. This is consistent with the findings of Song et al. [28], who reported that the viscosity of a lavender essential oil-based microemulsion with 60% water declined sharply with increasing temperature from 25 to 60 °C, while the viscosity of the microemulsion with 70% water content remained stable. This could be explained by the dependence of the microemulsion’s viscosity on water content. In another study, Kumar et al. [29] observed that the O/W microemulsions formulated with zwitterionic surfactant exhibited temperature thinning properties, i.e., their viscosity decreased with increasing temperature. The reduction in microemulsion viscosity with increasing temperature could be attributed to the increased mobility of surfactant at the oil–water interface, which resulted in the separation of surfactant aggregates and thus lowered the interfacial tension [30]. In the present study, the relatively low viscosity of 60% and 70% TaEO-MEs suggested that they were monodispersed O/W spherical droplets without any anisometric aggregates in the system.

### 3.4. The Antioxidant Activity of Pure and Microemulsified TaEO

**Figure 7:**
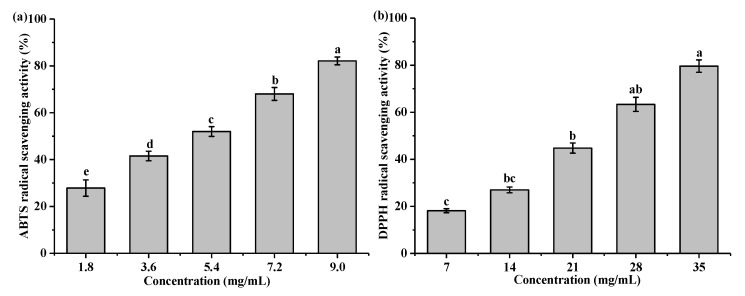
Antioxidant activities of *Torreya grandis* cv. aril essential oil in terms of ABTS (**a**) and DPPH (**b**) radical scavenging assays, respectively. Different superscripts within a row indicate significant differences (*p* < 0.05).

**Figure 8:**
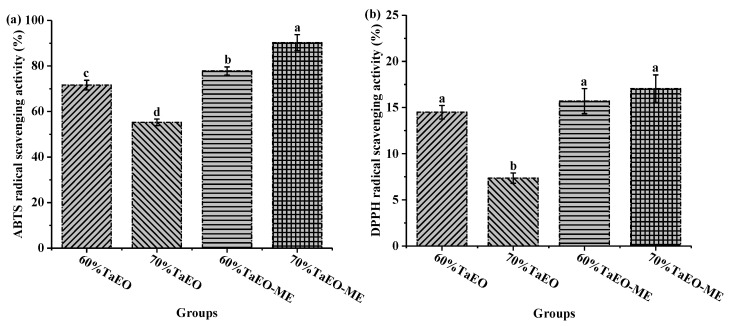
Antioxidant activities of TaEO and TaEO-ME in terms of ABTS (**a**) and DPPH (**b**) radical scavenging assays, respectively. 60% TaEO-ME = *Torreya grandis* cv. aril essential oil microemulsion with 60% water content; 70% TaEO-ME = *Torreya grandis* cv. aril essential oil microemulsion with 70% water content; 60% TaEO = an essential oil content equivalent to that of 60% water content microemulsion; 70% TaEO = an essential oil content equivalent to that of 70% water content microemulsion. Data within a row with different superscripts are significantly different (*p* < 0.05).

The antioxidant capacities of TaEO and TaEO-ME were evaluated by measuring their scavenging abilities toward ABTS and DPPH radicals. The results showed that TaEO exhibited a higher scavenging activity against ABTS radicals than DPPH radicals, with IC_50_ values of 4.14 ± 0.06 mg/mL and 20.73 ± 1.56 mg/mL, respectively (Figure 7). This could be due to the higher sensitivity of the ABTS method compared to the DPPH assay [31]. As shown in Figure 7, TaEO scavenged ABTS and DPPH radicals in a dose-dependent manner. Yu et al. [5] reported a similar DPPH radical scavenging activity for the essential oil from *Torreya grandis* cv. Merrillii aril, with an IC_50_ value of 17.13 mg/mL. Figure 8 shows that 70% TaEO-ME displayed a significantly higher ABTS radical scavenging capacity than 60% TaEO-ME. Moreover, both 60% and 70% TaEO-MEs had higher ABTS radical scavenging activity than TaEO at the same essential oil content (*p* < 0.05). For DPPH radical scavenging activity, there was no significant difference among 60% and 70% TaEO-MEs and 60% TaEO, but they were all higher than that of 70% TaEO (*p* < 0.05). These results demonstrated that microemulsion enhanced the antioxidant capacity of TaEO, which might be related to the improved solubility of TaEO in water and the larger specific surface area of TaEO-ME, facilitating the reaction of essential oil with free radicals [32]. The physical location of TaEO in the oil-in-water microemulsion can also influence its free radical scavenging activity [33]. The higher antioxidant effect of 70% TaEO-ME than 60% TaEO-ME might be explained by the lower concentration of surfactant micelles in the former system, which reduced the barrier for free radicals to access the essential oil [34]. On the other hand, the larger interfacial area of 70% TaEO-ME enabled more contact between the essential oil and the free radicals, leading to a higher scavenging activity [35]. 

### 3.5. The Tyrosinase Inhibition Activity of Pure and Microemulsified TaEO

**Figure 9:**
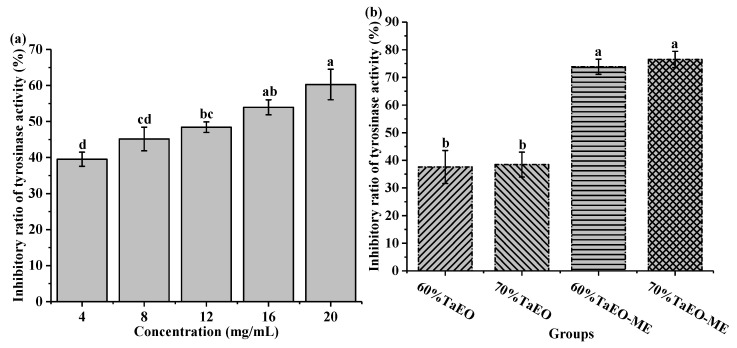
The tyrosinase inhibitory activity of TaEO (**a**) and TaEO-ME (**b**). 60% TaEO-ME = *Torreya grandis* cv. aril essential oil microemulsion with 60% water content; 70% TaEO-ME = *Torreya grandis* cv. aril essential oil microemulsion with 70% water content; 60% TaEO = an essential oil content equivalent to that of 60% water content microemulsion; 70% TaEO = an essential oil content equivalent to that of 70% water content microemulsion. Data within a row with different superscripts are significantly different (*p* < 0.05).

Tyrosinase is a key enzyme involved in mammalian melanogenesis and the enzymatic browning of fruit or vegetables [36]. Essential oils, as natural products, have received considerable attention for their ability to inhibit tyrosinase. The inhibition of tyrosinase by TaEO and TaEO-ME was determined, as shown in Figure 9. It was observed that TaEO showed a concentration-dependent inhibition of tyrosinase activity with an IC_50_ value of 11.04 ± 0.76 mg/mL (Figure 9a). This result was in agreement with a previous study, which reported that the essential oil from the peel of Chinese *Torreya grandis* Fort inhibited tyrosinase by 25.95% at 1.443 mg/mL [37]. Previous studies also revealed that some essential oils with citral, pinene, and D-limonene as their abundant compounds have demonstrated a potent tyrosinase inhibitory effect. For instance, lemon essential oil, which mainly consists of citral, β-pinene, and D-limonene, exhibited a strong tyrosinase inhibition [38]. Similarly, the essential oil of *Alpinia nantoensis*, which contains large amounts of D-limonene and α-pinene, showed remarkable anti-tyrosinase activity [39]. Thus, it is plausible that D-limonene and α-pinene, which were also the predominant constituents of TaEO, might account for the observed anti-tyrosinase effect of this essential oil.

Figure 9b illustrates that the tyrosinase inhibition of 60% and 70% TaEOs did not differ significantly, nor did that of the 60% and 70% TaEO-MEs (*p* > 0.05). The tyrosinase inhibition activity of 60% and 70% TaEOs was 38% and 39.5%, respectively. However, when TaEO was formulated into a microemulsion, the tyrosinase inhibition increased to 75% for 60% TaEO-ME and 70% for 70% TaEO-ME, using the same amount of essential oil as in 60% or 70% TaEOs. This result suggested that microemulsion enhanced the inhibitory effect of the essential oil. Zheng et al. [40] reported that norartocarpetin microemulsions maintained strong tyrosinase inhibition and anti-browning effects with good physicochemical stability for eight weeks. Another study found that the tyrosinase inhibitory activity of *Cinnamomum zeylanicum* essential oil was significantly increased using the microemulsion formulation [41]. Hence, based on previous and current data, the results of tyrosinase inhibition were consistent with those obtained in the DPPH and ABTS radical scavenging assays.

### 3.6. The Antibacterial Activity of Pure and Microemulsified TaEO

*S. aureus*, *L. monocytogenes*, *S. castellani*, *S. typhimurium*, and *E. coli* are food-borne pathogens that can cause serious health problems in humans. Essential oils derived from plants have been shown to possess antioxidant and antimicrobial activities, which could make them promising antimicrobial agents for controlling food-borne pathogenic and spoilage microorganisms in the food industry [42].

In the present study, the inhibition activity of TaEO and TaEO-ME against two Gram-positive bacteria, *S. aureus* and *L. monocytogenes*, and three Gram-negative bacteria, *S. castellani*, *S. typhimurium*, and *E. coli*, was investigated using the Oxford cup method. The diameters of the inhibition zone of TaEO against the tested strains are shown in Table 3. It was found that TaEO in free form was capable of inhibiting the growth of all five pathogenic bacteria tested. The largest inhibition zone was observed for the Gram-negative bacteria *S. castellani* (16.97 ± 0.54 mm), followed by the Gram-positive bacteria *S. aureus* (14.73 ± 0.62 mm). The inhibitory effect of TaEO was probably attributed to the predominant presence of D-limonene and α-pinene, which are oxygenated monoterpenes with well-known antibacterial properties [43]. Table 4 presents the results of the antimicrobial activity of 60% and 70% TaEOs and 60% and 70% TaEO-MEs against the selected bacterial strains. It was noted that mixed surfactants showed no antimicrobial effect (Table 4). However, at the same essential oil level, the inhibition zones of the TaEO-MEs (containing 60% or 70% water content) against all examined microorganisms were much greater than those of crude TaEO (*p* < 0.05), which indicated the microemulsion has an enhancing effect on the antimicrobial activity of TaEO. Similar observations have been reported in other studies, that microemulsification can improve the antibacterial activities of essential oils compared with their free counterparts [34,44,45]. Moreover, 60% TaEO-ME was more effective than 70% TaEO-ME against all five tested bacterial strains (*p* < 0.05), which may be presumably due to the dilution effect caused by a higher water content. One possible reason for the enhanced antimicrobial efficacy of TaEO is that microemulsion is a kinetically unstable system. When the microemulsion has an O/W structure, the aqueous phase acts as a continuous phase, allowing more droplets of this system to interact easily and directly with the bacterial cell wall. This may result in improved bacterial cell wall permeability induced by the surfactant and a higher penetration of essential oil across bacterial cell membranes [34,45]. In addition, the essential oil, whose water-solubility is enhanced by surfactant micelles [46], interacts strongly with the bacterial cell, reducing its membrane hydrophobicity and leading to a significant loss of bacterial viability [34,47]. Microemulsion is also reported to have a distorting effect on lipid conformation in the bilayer, which affects membrane fluidity and disrupts the plasma membrane, thus contributing to cell death [48].

### 3.7. Effects of TaEO and TaEO-ME Treatment on Loquat Fruit Preservation

#### 3.7.1. Decay Index, Ascorbic Acid, Total Phenolic Content, and MDA

**Figure 10:**
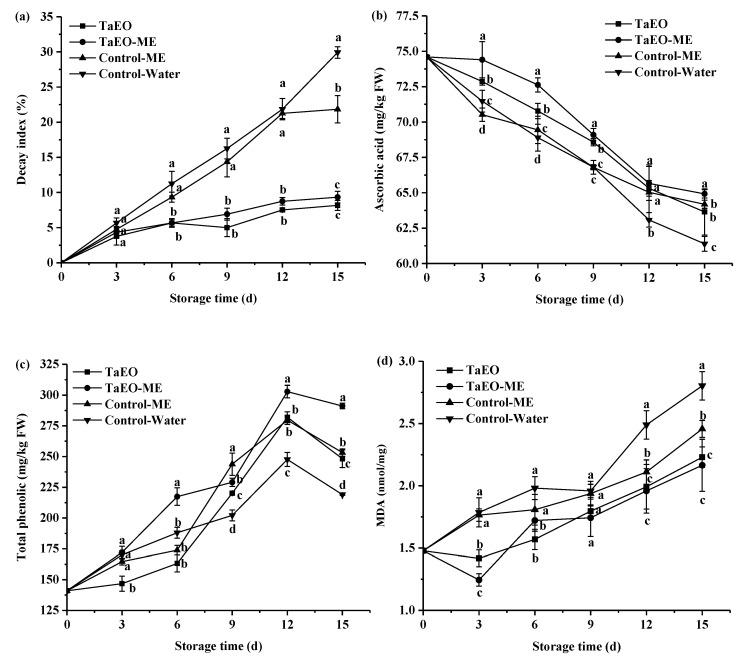
Changes in decay index (**a**), ascorbic acid (**b**), total phenolic (**c**), and MDA content (**d**) of loquat fruit during storage. Error bars represent the standard deviation (SD) of three replicates. Different lowercase letters within each panel indicate statistical significance between four groups at the same storage time.

As shown in Figure 10a, the decay index of loquat fruits in each treatment increased progressively during the storage period. By the 15th day, the control groups had a higher decay index of 30.00% and 21.88%, while the fruits treated with TaEO and TaEO-ME had a significantly lower decay index of 8.13% and 9.37%, respectively. This result indicates that TaEO and TaEO-ME treatments could effectively inhibit the decay of loquat fruit (*p* < 0.05), which could be ascribed to the antimicrobial activity of *Torreya grandis* essential oil on the fruit surface. Interestingly, no significant differences in decay index were found between the TaEO and TaEO-ME treatments throughout the storage period.

Ascorbic acid is an important indicator of the nutritional quality of loquat fruit. Figure 10b illustrates the changes in ascorbic acid content of each treatment during storage time. The ascorbic acid in both the control and treated fruits decreased gradually throughout the storage period, but the loquat fruits treated with TaEO and its microemulsion exhibited a slower rate of reduction. In addition, the highest ascorbic acid content was observed in the fruits treated with TaEO-ME during storage, which reached 64.92 mg/kg FW on the 15th day. These results suggest that the microemulsification of TaEO further enhances its efficacy in preserving the ascorbic acid content of loquat fruit. Our findings are consistent with previous studies that reported the beneficial effects of other essential oils on the ascorbic acid and other quality parameters of fruits and vegetables, such as pitaya fruit [49] and cucumber [50]. Ascorbate oxidase is the main enzyme responsible for the degradation of ascorbic acid. This enzyme increases its activity under stress conditions. Essential oils have antioxidant activity and can reduce the oxidative stress in plant tissues. Thus, the gradual release of TaEO from TaEO-ME may enhance the antioxidant activity of essential oil for a longer period by protecting it from degradation by oxygen, light, and temperature, which may result in less degradation of ascorbic acid in treated loquat fruit.

Loquat fruit also contains various phytochemicals that exhibit antioxidant activity and modulate the quality attributes of the fruit, such as phenolic compounds and flavonoids [51]. These phytochemicals may also influence the sensory properties of the fruit, such as texture, flavor, and taste. The total phenolic content of loquat fruit under different treatments during storage is shown in Figure 10c. The total phenolic content increased slowly in the first 6 days of storage, reached the peak on the 12th day, and then declined. This trend is in agreement with recent studies which have reported that nano SiO_2_ or serine protease treatment enhanced the total phenolic content of loquat fruit in the early stage of storage, followed by a decrease in the later stage under ambient conditions [1,14]. Moreover, the content of total phenolic in loquat fruit treated with TaEO-ME was higher than that of the other groups from the 12th day to 15th day. Essential oils are hypothesized to act as elicitors that induce a priming effect in plants, enhancing their resistance to biotic and abiotic stresses [52]. Thus, the treatment of loquat fruits with TaEO-ME might have stimulated the biosynthesis of phenolic compounds as a part of the plant’s defense system.

MDA is a terminal product of lipid peroxidation in biological membranes, and its concentration can serve as an indicator of the degree of membrane damage. Figure 10d shows that the MDA content of loquat fruits increased over storage time in all treatments, indicating that the membrane integrity was gradually compromised. However, TaEO and TaEO-ME treatments effectively delayed the accumulation of MDA in the flesh of loquat fruit throughout the storage period, especially from the 12th to the 15th day. At the end of the storage period, the MDA content in TaEO- and TaEO-ME-treated fruits was 2.23 and 2.16 nmol/mg, respectively, which was 20.48% and 22.76% lower than that in the Control-Water group. These results suggested that TaEO and TaEO-ME treatments enhanced the activities of defense-related enzymes that scavenge reactive oxygen species (ROS) [53], thereby protecting the membrane from oxidative damage.

#### 3.7.2. Activities of Defense-Related Enzymes in Loquat Fruit

**Figure 11:**
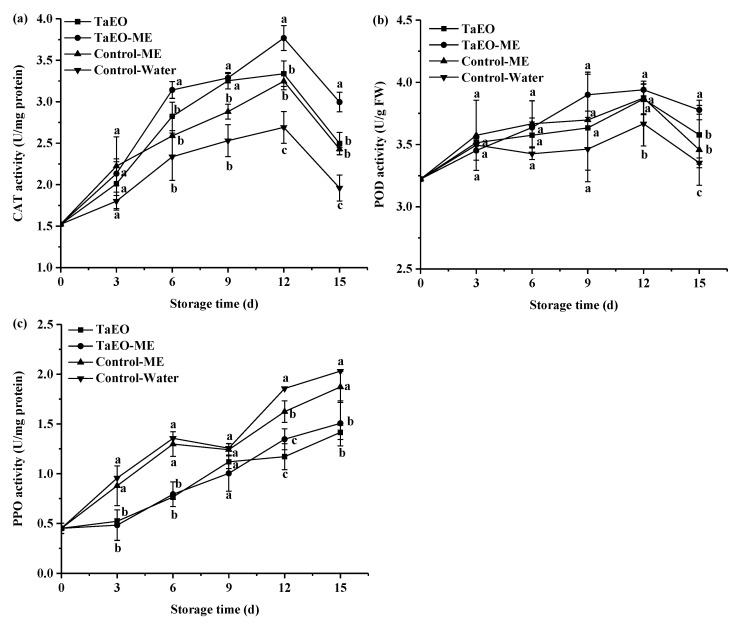
Changes in activities of CAT (**a**), POD (**b**), and PPO (**c**) during loquat fruit storage. Error bars represent the standard deviation (SD) of three replicates. Different lowercase letters within each panel indicate significant significance among the four groups at the same storage time.

CAT, POD, and PPO are the most studied defense-related enzymes. These are also important biochemical indicators of fruit quality during postharvest storage and processing. Loquat fruit is prone to flesh browning, which is mediated by PPO and POD enzymes, which oxidize endogenous phenolic compounds. The activities of CAT, POD, and PPO enzymes in loquat fruit under different treatments are presented in Figure 11. CAT is a key enzyme in the plant antioxidant system, which catalyzes the decomposition of hydrogen peroxide into water and oxygen. With the extension of storage time, the CAT activity of all samples increased until day 12, and then decreased (Figure 11a), but the fruits treated with coating maintained CAT activity at a relatively high level. This suggested that coating could increase the activity of CAT in loquat fruit, and the treatment with microemulsified TaEO had the best effect. This could be due to the microemulsification of TaEO, which might effectively activate the CAT enzyme system of loquat fruit. A similar effect of essential oils on CAT activity was reported for cherry tomato fruit [54].

POD is a key enzyme involved in the oxidative stress-induced enzymatic defense system [55]. Like CAT, it catalyzes the disproportionation of H_2_O_2_ to water and oxygen. It can also oxidize phenolic substrates, thereby removing both H_2_O_2_ and phenolic toxicity from plants. The activity of POD showed a similar pattern to that of CAT. As illustrated in Figure 11b, the POD activity in both the control and treated fruits increased gradually and reached its peak level after 12 days of storage. Moreover, the fruit treated with TaEO-ME had the highest POD activity at the late storage period, indicating that the microemulsification of TaEO could improve POD activity in loquat fruit more effectively, and alleviate the oxidative damage to the cells. This is consistent with previous research, which reported enhanced POD activity in pitaya fruit treated with essential oil *p*-anisaldehyde [49].

Our study demonstrated that TaEO-ME treatment increased the activities of CAT and POD, two key antioxidant enzymes, in loquat fruit. This effect was associated with a reduction in ROS accumulation, which is a major cause of oxidative stress and senescence in fruits. The coordinated action of CAT and POD may enhance the oxidation resistance of loquat fruit, thereby preserving its quality and extending its shelf life. These findings suggest that the TaEO-ME treatment could effectively reduce the ROS level and lipid peroxidation of loquat fruit by inducing increased antioxidant activity [14].

PPO is an enzyme that catalyzes the oxidation of endogenous phenolic substances, which are abundant in fruits, leading to senescence and browning phenomena [56]. Loquat fruit has a soft texture and is susceptible to spoilage. When the cells of loquat fruit are damaged, intracellular compartments are disrupted, resulting in the release of phenolics from the vesicles and an increase in PPO activity. Figure 11c shows the changes in the PPO activity of loquat fruits during storage. PPO activity increased steadily in all samples, and reached its peak on the last day of storage. However, the loquat fruit treated with TaEO and TaEO-ME had significantly lower PPO activity than the control group throughout the storage period. There was no significant difference in PPO activity between the fruits treated with TaEO and TaEO-ME. This suggests that TaEO may exert an inhibitory effect on PPO activity, as reported previously in tomato fruit [57].

## 4. Conclusions

In conclusion, this study demonstrated that the essential oil extracted from the *Torreya grandis* cv. Merrillii aril can be used as a natural preservative for loquat fruit by forming a stable and biologically active microemulsion system. The TaEO-ME formulations with 60% and 70% water content showed superior physicochemical and biological properties compared to free TaEO, such as a smaller droplet size, higher stability, and enhanced antioxidant, anti-tyrosinase, and antibacterial activities. The TaEO-ME treatment also improved the postharvest quality of loquat fruit by reducing the decay index and membrane lipid peroxidation, preserving the nutrient content, and modulating enzyme activities. Therefore, TaEO-ME can be considered a promising alternative to chemical preservatives for extending the shelf life of loquat fruit.

## Figures and Tables

**Table 1:** Effect of surfactant/TaEO mass ratio on TaEO-ME formation.

*S_m_*	Particle Size (nm)	PDI	ζ-Potential (mV)
4:1	143.90 ± 2.34 ^a^	0.79 ± 0.01 ^b^	−0.04 ± 0.08 ^a^
5:1	109.83 ± 1.33 ^b^	1.00 ± 0 ^a^	−1.04 ± 0.16 ^b^
6:1	13.00 ± 0.032 ^c^	0.26 ± 0 ^c^	−6.35 ± 1.77 ^c^
7:1	12.38 ± 0.13 ^c^	0.24 ± 0.02 ^c^	−6.61 ± 2.15 ^c^
8:1	11.67 ± 0.32 ^c^	0.19 ± 0.03 ^c^	−7.26 ± 3.03 ^c^
9:1	11.36 ± 0.13 ^c^	0.19 ± 0.02 ^c^	−6.67 ± 0.61 ^c^

Note: Data within each row that have different superscripts indicate significant differences (*p* < 0.05).

**Table 2:** Particle size, PDI, and ζ-potential of 60%TaEO-ME and 70%TaEO-ME after storage for 180 days.

Storage Time (d)	Water Content	Particle Size (nm)	PDI	ζ-Potential (mV)
0	60% TaEO-ME	11.36 ± 0.21 ^a^	0.20 ± 0.02 ^b^	−0.21 ± 0.05 ^a^
	70% TaEO-ME	11.15 ± 0.10 ^a^	0.24 ± 0.01 ^a^	−0.45 ± 0.24 ^a^
180	60% TaEO-ME	11.73 ± 0.41 ^a^	0.15 ± 0.02 ^b^	−1.50 ± 0.85 ^b^
	70% TaEO-ME	11.33 ± 0.11 ^a^	0.22 ± 0.02 ^a^	−1.72 ± 0.19 ^b^

Note: 60% TaEO-ME and 70% TaEO-ME represent the microemulsions of TaEO with water contents of 60% and 70%, respectively. The data within each row with different superscripts are significantly different (*p* < 0.05).

**Table 3:** Antibacterial activity of TaEO against selected bacterial strains.

Pathogens Tested	Inhibition Zone Diameter (mm)
*Staphylococcus aureus* (G^+^)	14.73 ± 0.62
*Listeria monocytogenes* (G^+^)	13.13 ± 0.58
*Shigella castellani* (G^−^)	16.97 ± 0.54
*Salmonella typhimurium* (G^−^)	13.51 ± 0.27
*Escherichia coli* (G^−^)	12.76 ± 0.26

**Table 4:** Antibacterial activity of TaEO and its microemulsions against tested bacteria.

	Inhibition Zone Diameter (mm)				
Pathogens Tested	Mixed Surfactants	60% TaEO-ME	70% TaEO-ME	60% TaEO	70% TaEO
*Staphylococcus aureus* (G^+^)	ND	11.34 ± 0.25 ^a^	10.62 ± 0.56 ^b^	9.17 ± 0.06 ^c^	8.61 ± 0.31 ^d^
*Listeria monocytogenes* (G^+^)	ND	11.99 ± 0.37 ^a^	10.72 ± 0.64 ^b^	8.10 ± 0.04 ^c^	8.06 ± 0.01 ^c^
*Shigella castellani* (G^−^)	ND	14.09 ± 1.04 ^a^	11.78 ± 0.10 ^b^	9.25 ± 0.49 ^c^	8.05 ± 0.01 ^d^
*Salmonella typhimurium* (G^−^)	ND	12.52 ± 2.67 ^a^	11.20 ± 1.51 ^b^	8.17 ± 0.06 ^c^	8.11 ± 0.01 ^c^
*Escherichia coli* (G^−^)	ND	10.74 ± 0.82 ^a^	8.97 ± 0.49 ^b^	8.06 ± 0.01 ^c^	8.04 ± 0.11 ^c^

Note: 60% TaEO-ME = *Torreya grandis* cv. aril essential oil microemulsion with 60% water content; 70% TaEO-ME = *Torreya grandis* cv. aril essential oil microemulsion with 70% water content; 60% TaEO = an essential oil amount equivalent to that of 60% TaEO-ME; 70% TaEO = an essential oil amount equivalent to that of 70% TaEO-ME; ND = not detected. Data within a row with different superscripts are significantly different (*p* < 0.05).

## Data Availability

All the data presented in this study are available within the article.
